# Are There Any Differences in Abdominal Activation between Women and Men during Hypopressive Exercises?

**DOI:** 10.3390/ijerph18136984

**Published:** 2021-06-29

**Authors:** Iria Da Cuña-Carrera, Alejandra Alonso-Calvete, Eva M. Lantarón-Caeiro, Mercedes Soto-González

**Affiliations:** 1 Facultade de Fisioterapia, Universidade de Vigo, 36310 Pontevedra, Spain; iriadc@uvigo.es (I.D.C.-C.); alejalonso@uvigo.es (A.A.-C.); m.soto@uvigo.es (M.S.-G.); 2 Facultade de Ciencias da Educación e do Deporte, Universidade de Vigo, 36310 Pontevedra, Spain

**Keywords:** abdominal muscles, hypopressive exercises, sex differences, ultrasonography

## Abstract

This study analyzes the effects of hypopressive exercises on the abdominal thickness of healthy subjects and compares the performance between women and men. We conducted a transversal observational study in 98 subjects (63% women). The muscle thickness is analyzed in transversus abdominis, internal oblique, external oblique, and rectus abdominis with ultrasound imaging at rest and during the hypopressive exercise (HE) in supine and standing position. Comparisons between rest and hypopressive exercise are carried out in the two different positions and between women and men. In the supine position, there is a significant activation of the transversus abdominis and internal oblique during hypopressive exercise (*p* < 0.001), and it is similar in both sexes, the external oblique is only activated significantly by men (*p* < 0.001) and rectus abdominis had no significant activation (*p* > 0.05). Our results show that standing transversus abdominis and external oblique significantly increased their thickness during HE with higher effects in men. Internal oblique also increased significantly, but with higher effects in women, and rectus abdominis had no significant increase. Men had similar effects to women during HE, with an activation of the deepest abdominal muscles. The unequal anatomy and the position could explain the different results obtained between the sexes.

## 1. Introduction

Hypopressive exercises (HE) are described as postural techniques performed through breathing in combination with apnea in different positions [1]. These exercises are demonstrated to decrease intraabdominal pressure and activate the pelvic floor muscles and the abdominal wall, and thus, they are designed for the prevention and treatment of perineal dysfunctions, especially in postpartum [1]. Consequently, HE are mainly performed by women, with benefits in pelvic floor dysfunctions or urinary incontinence [2,3,4,5,6,7,8], but recently new scientific research has demonstrated that men with urinary incontinence, scoliosis, or low back pain could also be benefited from these exercises [9,10,11,12]. Other studies have shown that HE could induce changes in postural muscles, such as increases in muscle thickness, cross-sectional area, or length [10,11,12]. These modifications could influence the low back, but also the pelvic floor or the abdominal muscles [13], with implications in the biomechanics of balance, gait, and posture [14,15]. However, the postural muscles have anatomical differences according to sex [16]. Specifically, the internal and external oblique in the abdominal wall of men continue with the cremaster muscle and cremaster fascia, but in women, this area does not exist, and the fascia of the obliques continues with the round ligament [16]. For this reason, HE could have different effects on the abdominal muscles between men and women, but there is a lack of knowledge in this sense and especially in the effects of HE in men, as most studies included only women in their samples [17,18,19,20]. Consequently, the training programs and rehabilitation processes with HE could be influenced by sex, and the effectiveness of this exercise could be conditioned to the different anatomy or different thickness of the abdominal wall.

Therefore, this study analyzes the effects of HE on the abdominal wall in healthy subjects of both sexes, and to compare the performance of the HE between women and men.

## 2. Materials and Methods

### 2.1. Participants and Design of the Study

A transversal observational study was conducted to analyze the effects of HE on muscle thickness according to sex, measured by ultrasound imaging.

The sample size was based on data from Amerijckx et al. [21] using the variable “transversus abdominis thickness (average) at the end of relaxed expiration” (4.1 ± 1.4 cm) versus “transversus abdominis thickness (average) at the end of full expiration” (5.9 ± 2.1 cm). G*Power software 3.1 was used with a power of 0.95, and an α error of 0.05, and a sample of 13 subjects was estimated. Inclusion criteria were men and nulliparous women, and exclusion criteria were pregnancy, abdominal surgery or abdominal muscle disease, arterial hypertension, and neurological or autoimmune disorders [6,11,17]. First, 100 subjects were included, and after applying the inclusion and exclusion criteria, two participants were discarded for have given birth. Finally, 98 subjects (64% women, 36% men; age 22.43 ± 3.56 years; body mass index 22.81 ± 2.75 kg/m^2^) were voluntarily recruited. All participants were healthy students with no history of chronic breathing diseases.

All subjects received oral and written information about the study and signed written informed consent. Ethical approval was granted by the Ethics Committee of the Faculty of Physiotherapy (University of Vigo) with the number 17,072,018, and the principles of the Declaration of Helsinki (2004) were followed.

### 2.2. Procedures

Before the intervention, participants received two familiarization sessions of training with HE, to perform the intervention correctly, with one week between both sessions. These sessions were carried out by an expert and qualified physical therapist and supervised by other researchers. The first session consisted of explaining the HE, asking subjects to do a spine elongation with the neutral pelvis and scapular muscle activation. In that position, participants were asked to perform three normal breathing cycles following by a slow deep exhalation in the last breath. After the exhalation, they were asked to keep a breath-holding with expansion and lift of the ribs [1]. At the end of the first session, a researcher checked the performance of the HE. In the second familiarization session, subjects who did not perform the HE correctly repeated the learning program. Finally, all subjects achieved a good level of HE and were included in the measurements.

### 2.3. Task Performance

The muscle thickness of the abdominal wall was measured in two situations: (1) At rest before the HE and (2) during HE, to analyze the activation of the muscle during this exercise. In addition, the exercises were performed in two different positions: supine and standing. All measurements were performed with ultrasound imaging, using a 5–10 MHz lineal ultrasounds transduce (Fujifilm SonoSite M-Turbo^®^, Bothell, WA, USA). A physical therapist with knowledge and training in ultrasound imaging of the abdominal muscles conducted the measurements. For rectus abdominis, the probe was located on the anterior abdominal wall, laterally to the navel [22]. For external oblique, internal oblique, and transversus abdominis, the probe was located laterally between the iliac crest and rib cage [13,19]. All measurements were performed once and in a randomized order, and 5 min were waited between exercises to avoid fatigue. All measurements were performed on the dominant side of the subject. Once the muscle was located, measurements of the thickness were recorded using the on-screen Calipper, perpendicular to the hyper-echoic area of the muscle and between fascial borders [22,23].

### 2.4. Statistical Analysis

All analyses were conducted using the statistical package SPSS for Macintosh (version 25.0. Armonk, NY, USA: IBM Corp). The normality of distribution for each variable was checked both graphically and using the Kolmogorov-Smirnov test. The descriptive results of these variables are presented by mean ± standard deviation (SD). A T-Student test was carried out to analyze sex differences in body mass index (BMI). A repeated-measures ANOVA was used to analyze sex differences in abdominal thickness with two intra-subject factors: Situation (rest and HE) and position (supine or standing). Pair-wise comparisons were conducted via a Bonferroni posthoc test, using Cohens′ d to calculate effect sizes [24]. These effects were classified as trivial (d < 0.2) small (0.2 < d < 0.5), medium (0.5 < d < 0.8), and large (d ≥ 0.8) [24]. For all analyses, the significance value was set at *p* ≤ 0.05.

## 3. Results

Descriptive data of the subjects are shown in Table 1 according to sex.

**Figure 1:**
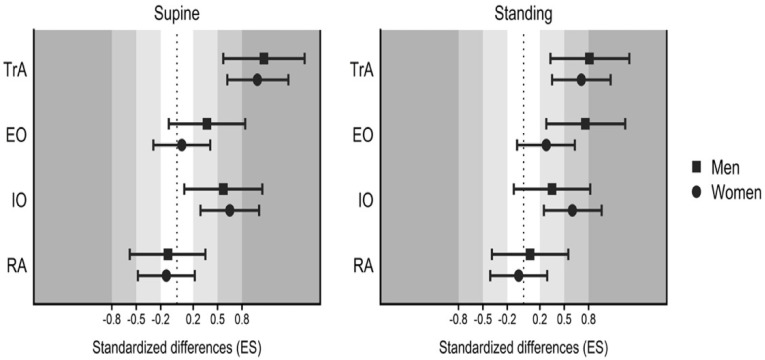
Standardized differences (Cohens′ d) in abdominal thickness between rest and hypopressive exercise in both sexes in supine (**left**) and standing (**right**). TrA, transversus abdominis; IO, internal oblique; EO, external oblique; RA, rectus abdominis.

Participants in this study had similar characteristics, with no significant differences in age or BMI between sexes (*p* > 0.05). Regarding the thickness, results of the comparisons between rest and HE in both sexes are shown in Table 2 for the supine position and in Table 3 for the standing position. Moreover, Figure 1 depicts the standardized differences in abdominal thickness between rest and HE in both sexes.

As described in Table 2 and Table 3, TrA and IO significantly increased their thickness during HE compared to rest, with no differences between the position or sex (*p* > 0.05). However, in EO, there were significant differences according to the position (*p* < 0.01), since in standing, there was a significant increase in the muscle thickness for both women (*p* = 0.023) and men (*p* < 0.001), but in supine only men had significant differences during HE (*p* = 0.047). Finally, RA did not increase with HE in any sex or position (*p* > 0.05).

In the pair-wise comparisons between sexes (Figure 1), results showed that in TrA both women (d = 0.99, large) and men (d = 1.07, large) had great effects with HE in supine position, however, in standing men had higher effects with HE (d = 0.81, large) than women (d = 0.71, medium). In the IO, both women (d = 0.65, medium) and men (d = 0.57, medium) has similar effects with HE in supine, but in standing women (d = 0.6, medium) had higher effects with HE than men (d = 0.35, small). Regarding the EO, only men increased the thickness with HE in supine (d = 0.37, small), but when standing, both sexes increase the thickness, with higher effects in men (d = 0.76, medium) compared to women (d = 0.28, small).

## 4. Discussion

The aim of this study was to assess the effects of HE on the abdominal muscles of healthy subjects of both sexes, comparing these effects between women and men and in the supine and standing positions. The main results of this investigation suggested that both women and men activated their TrA, IO, and EO during HE, but the position and sex influenced this activation.

Previous research has demonstrated significant differences in abdominal muscles′ thickness between women and men, as reported in this study [25]. These differences could be due to the unequal anatomy of this area between sexes [16], but also to the different BMI [25]. The findings presented suggest that BMI does not influence the abdominal thickness in this sample, since there are no significant differences between sexes in this variable. Furthermore, the differences in the abdominal activation between sexes have also been described with similar exercises [26], and men always reported higher thickness than women. For this reason, all changes made on the abdominal muscles during HE could be explained by many factors, such as the HE, sex, or position.

In this regard, during the supine position, the four abdominal muscles were assessed, showing that TrA and IO increase their thickness significantly during HE compared to rest in both sexes. Comparing the effects of HE between women and men in these muscles, it has been shown that there were no differences between the sexes in this position. In EO, only men significantly increased the thickness with small effects, and in RA, there was no increase in women or men. However, assessing these muscles in standing results reported that TrA and IO increased their thickness significantly during the HE, but in TrA men, had a larger increase than women and in IO women had a larger increase than men. In the EO in standing both, sexes increased the thickness during HE, but men had higher effects than women, and in RA, there were no significant differences in the thickness between rest and HE. These results are consistent with prior investigations reporting that HE generate the activation of the abdominal muscles, especially the deepest ones [19,20,27]. During these exercises, an involuntary contraction of the TrA and the pelvic floor muscles is performed [28], and it has been demonstrated that this contraction could activate the other abdominal muscles, due to the fascial connections throughout the abdominal wall [23]. However, these connections depend on the muscle, since the TrA and IO have a closer relationship than the TrA and EO or RA, and on the anatomical differences between women and men [16,22,23]. In this sense, the activation of the abdominal muscles between sexes has never been studied, although the different performance of abdominal exercises between sexes has been described [28,29,30], stating that there are no differences between sexes in terms of neuromuscular control or the ability of contraction [31]. Nevertheless, the anatomical differences between sexes have been clearly defined, specifically in the urogenital area where the different viscera and muscles create different relationships among them. Therefore, one of the main hypotheses of the different results presented in this study according to sex is the different disposition of the pelvic floor muscles and pelvic viscera, which could influence the deepest abdominal muscles differently [16].

On the other hand, the results of this study are undoubtedly influenced by the position during HE. Abdominal exercises and specifically HE have been largely studied in both positions, demonstrating that the contraction of the TrA and the synergy between abdominal muscles appeared despite the position [27,28,29]. However, in a more in-depth analysis of each muscle, several differences should be considered. First, the standing position requires activation of the abdominal wall, but also other muscles, such as multifidous, quadratus lumborum, or even the thoracolumbar fascia [32,33]. Thus, this position could activate the fascial connections between these structures and the abdominal muscles, generating a pre-activation before the exercise [33]. This hypothesis is consistent with findings in this study, since the thickness of each abdominal muscle in standing during rest is larger than in supine during rest, suggesting a contraction of the abdominal wall prior to the exercise and due to the position. Moreover, the anatomical differences between sexes could play an important role in this analysis, since the gravity act more in standing than in supine, and the weight of the unequal pelvic viscera could influence the contraction of the abdominal muscles. Accordingly, the TrA and IO, the deepest abdominal muscles, are more influenced by the HE [17,18,19,27], and they present similar results despite the position, but HE has demonstrated to influence less the EO [34], and the position appears to play an important role in its activation. Finally, RA did not show any significant changes during HE in any position or according to sex, supporting the theory that HE influences more the deepest abdominal, and the position has no effects in this sense.

### 4.1. Limitations

Findings in this investigation are clearly conditioned by the lack of studies analyzing sex differences when performing abdominal exercises, but their importance is remarkable, since dysfunctions on the pelvic floor or abdominal area are presented in both sexes, but the differences in the activation have never been studied. Several hypotheses have been considered, but indeed some limitations are reflected. First, the thickness of the abdominal muscles was measured by ultrasound imaging, but previous studies used other methods, such as surface electromyography, which could provide different results, due to the depth of the examination. Likewise, in this study, only the muscle thickness was measured, but other architectural variables, such as the cross-sectional area or the muscle fascicle length, could complete the results provided in this study. Finally, there are some variables that have not been measured in this study, since only healthy subjects were included, but could be considered in future research, such as the lumbar lordosis or low back pain, which could influence the muscle fibers and generate an unequal activation of the abdominal muscles.

### 4.2. Practical Applications

To the best of the authors′ knowledge, this is the first study analyzing the contraction of the abdominal muscles during HE in both sexes and considering sex differences. Up to now, the benefits of HE have been studied and described in women with pelvic floor or abdominal dysfunctions; however, the results presented in this study provide for the first time information about the activation of the abdominal muscles in men during HE, and support their use in several situations, such as urinary incontinence after prostatectomy or pelvic floor muscles′ dysfunctions, where men and women need similar treatments, but from different perspectives, according to their anatomy and also to the activation of their muscles [12,35,36].

## 5. Conclusions

Hypopressive exercises activate the TrA, IO, and EO in women and men, but with differences according to sex or position.

## Figures and Tables

**Table 1:** Participants′ characteristics mean (SD).

	Women (n = 63)	Men (n = 35)	*p*-Value
Age (years)	22.34(3.69)	22.6(3.36)	0.208
Weight (kg)	60.61(8.3)	73.53(8.25)	0.072
Height (cm)	164.38(5.75)	177(6.15)	0.015
Body mass index	22.13(5.09)	22.82(2.68)	0.378

**Table 2:** Comparison between rest and HE in both sexes in supine mean (SD).

	Women			Men		
	Rest	HE	Δ (%)	Rest	HE	Δ (%)
TrA	0.29(0.09)	0.39(0.11) *	34.45	0.38(0.1)	0.57(0.23) *	50
IO	0.63(0.15)	0.76(0.24) *	20.64	1.01(0.29)	1.19(0.34) *	17.82
EO	0.52(0.15)	0.53(0.19)	1.92	0.70(0.19)	0.78(0.24) *	11.43
RA	0.93(0.17)	0.91(0.13)	−2.15	1.23(0.18)	1.21(0.17)	−1.63

* Significant difference (*p* < 0.05) between rest and HE. TrA, transversus abdominis; IO, internal oblique; EO, external oblique; RA, rectus abdominis.

**Table 3:** Comparison between rest and HE in both sexes in standing (mean ± SD).

	Women			Men		
	Rest	HE	Δ (%)	Rest	HE	Δ (%)
TrA	0.42(0.11)	0.53(0.19) *	26.19	0.53(0.14)	0.68(0.22) *	28.3
IO	0.65(0.2)	0.79(0.26) *	21.54	1.08(0.35)	1.21(0.39) *	12.04
EO	0.53(0.14)	0.57(0.15) *	7.55	0.61(0.16)	0.77(0.25) *	26.23
RA	0.93(0.17)	0.92(0.16)	−1.08	1.17(0.29)	1.19(0.19)	1.71

* Significant difference (*p* < 0.05) between rest and HE. TrA, transversus abdominis; IO, internal oblique; EO, external oblique; RA, rectus abdominis.

## References

[1] Caufriez M. (1997). Gymnastique Abdominale Hypopressive.

[2] Bernardes B.T., Resende A.P.M., Stüpp L., Oliveira E., Castro R.A., Jármy di Bella Z.I.K., Girão M.J.B.C., Sartori M.G.F. (2012). Efficacy of pelvic floor muscle training and hypopressive exercises for treating pelvic organ prolapse in women: Randomized controlled trial. Sao Paulo Med. J..

[3] Resende A.P.M., Stüpp L., Bernardes B.T., Oliveira E., Castro R.A., Girão M.J.B.C., Sartori M.G.F. (2012). Can hypopressive exercises provide additional benefits to pelvic floor muscle training in women with pelvic organ prolapse?. Neurourol. Urodyn..

[4] Juez L., Núñez-Córdoba J.M., Couso N., Aubá M., Alcázar J.L., Mínguez J.Á. (2019). Hypopressive technique versus pelvic floor muscle training for postpartum pelvic floor rehabilitation: A prospective cohort study. Neurourol. Urodyn..

[5] Navarro-Brazález B., Prieto-Gómez V., Prieto-Merino D., Sánchez-Sánchez B., McLean L., Torres-Lacomba M. (2020). Effectiveness of Hypopressive Exercises in Women with Pelvic Floor Dysfunction: A Randomised Controlled Trial. J. Clin. Med..

[6] Resende A.P.M., Bernardes B.T., Stüpp L., Oliveira E., Castro R.A., Girão M.J., Sartori M.G. (2019). Pelvic floor muscle training is better than hypopressive exercises in pelvic organ prolapse treatment: An assessor-blinded randomized controlled trial. Neurourol. Urodyn..

[7] Costa T., Resende A., Seleme R., Stüpp L., Castro R., Berghmans B. (2011). Hypopressive Gimnastics as a Resource for Perineal Propioception in Women with Urinary Incontinence. Fisioter. Bras..

[8] Rial T., Chulvi-Medrano I., Cortell-Tormo J., Alvárez-Sáez M. (2015). Puede un programa de ejercicio basado en técnicas hipopresivas mejorar el impacto de la incontinencia urinaria en la calidad de vida de la mujer?. Suelo. Pélvico..

[9] Au D., Matthew A.G., Alibhai S.M., Jones J.M., Fleshner N.E., Finelli A., Elterman D., Singal R.K., Jamnicky L., Faghani N. (2020). Pfilates and Hypopressives for the Treatment of Urinary Incontinence After Radical Prostatectomy: Results of a Feasibility Randomized Controlled Trial. PMR.

[10] Rami-Colás C., Martín-Nogueras A. (2016). Physiotherapy treatment of idiopathic scoliosis: Schorth versus hypopressive gymnastics. Fisioterapia.

[11] Bellido-Fernández L., Jiménez-Rejano J.J., Chillón-Martínez R., Gómez-Benítez M.A., De-La-Casa-Almeida M., Rebollo-Salas M. (2018). Effectiveness of Massage Therapy and Abdominal Hypopressive Gymnastics in Nonspecific Chronic Low Back Pain: A Randomized Controlled Pilot Study. Evid. Based Complement. Alternat. Med..

[12] González M.S., Carrera I.D.C., Nieto M.G., García S.L., Calvo A.O., Caeiro E.M.L. (2020). Early 3-month treatment with comprehensive physical therapy program restores continence in urinary incontinence patients after radical prostatectomy: A randomized controlled trial. Neurourol. Urodyn..

[13] Resende A.P.M., Torelli L., Zanetti M.R.D., Petricelli C.D., Jármy-Di Bella Z.I.K., Nakamura M.U., Júnior E.A., Moron A.F., Girão M.J.B.C., Sartori M.G.F. (2016). Can Abdominal Hypopressive Technique Change Levator Hiatus Area? A 3-Dimensional Ultrasound Study. Ultrasound Q..

[14] Lee D.G., Lee L.J., McLaughlin L. (2008). Stability, continence and breathing: The role of fascia following pregnancy and delivery. J. Bodyw. Mov. Ther..

[15] Ekstrom R.A., Donatelli R.A., Carp K.C. (2007). Electromyographic analysis of core trunk, hip, and thigh muscles during 9 rehabilitation exercises. J. Orthop. Sports Phys. Ther..

[16] Moore K., Dalley A.F., Agur M.R. (2017). Clinically Oriented Anatomy.

[17] Stüpp L., Resende A.P.M., Petricelli C.D., Nakamura M.U., Alexandre S.M., Zanetti M.R.D. (2011). Pelvic floor muscle and transversus abdominis activation in abdominal hypopressive technique through surface electromyography. Neurourol. Urodyn..

[18] Ithamar L., de Moura Filho A.G., Rodrigues M.A.B., Cortez K.C.D., Machado V.G., de Paiva Lima C.R.O., Moretti E., Lemos A. (2018). Abdominal and pelvic floor electromyographic analysis during abdominal hypopressive gymnastics. J. Bodyw. Mov. Ther..

[19] Navarro Brazález B., Sánchez Sánchez B., Prieto Gómez V., De La Villa Polo P., McLean L., Torres Lacomba M. (2020). Pelvic floor and abdominal muscle responses during hypopressive exercises in women with pelvic floor dysfunction. Neurourol. Urodyn..

[20] Navarro-Brazález B., Torres-Lacomba M., Arranz-Martín B., Sánchez-Mémdez O. (2017). Muscle response during a hypopressive exercise after pelvic floor physiotherapy: Assessment with transabdominal ultrasound. Fisioterapia.

[21] Amerijckx C., Goossens N., Pijnenburg M., Musarra F., van Leeuwen D.M., Schmitz M., Janssens L. (2020). Influence of phase of respiratory cycle on ultrasound imaging of deep abdominal muscle thickness. Musculoskelet. Sci. Pract..

[22] Teyhen D.S., Miltenberger C.E., Deiters H.M., Del Toro Y.M., Pulliam J.N., Childs J.D., Boyles R.E., Flynn T.W. (2005). The use of ultrasound imaging of the abdominal drawing-in maneuver in subjects with low back pain. J. Orthop. Sports Phys. Ther..

[23] Teyhen D.S., Gill N.W., Whittaker J.L., Henry S.M., Hides J.A., Hodges P. (2007). Rehabilitative ultrasound imaging of the abdominal muscles. J. Orthop. Sports Phys. Ther..

[24] Cohen J. (1988). Statistical Power Analysis for the Behavioral Sciences.

[25] Rankin G., Stokes M., Newham D.J. (2006). Abdominal muscle size and symmetry in normal subjects. Muscle Nerve.

[26] Manshadi F.D., Parnianpour M., Sarrafzadeh J., Azghani M.R., Kazemnejad A. (2011). Abdominal hollowing and lateral abdominal wall muscles’ activity in both healthy men & women: An ultrasonic assessment in supine and standing positions. J. Bodyw. Mov. Ther..

[27] del Mar Moreno-Muñoz M., Hita-Contreras F., Estudillo-Martínez M.D., Aibar-Almazán A., Castellote-Caballero Y., Bergamin M., Gobbo S., Cruz-Díaz D. (2021). The Effects of Abdominal Hypopressive Training on Postural Control and Deep Trunk Muscle Activation: A Randomized Controlled Trial. Int. J. Environ. Res. Public Health.

[28] Ainscough-Potts A.-M., Morrissey M.C., Critchley D. (2006). The response of the transverse abdominis and internal oblique muscles to different postures. Man. Ther..

[29] Sapsford R.R., Hodges P.W. (2012). The effect of abdominal and pelvic floor muscle activation on urine flow in women. Int. Urogynecol. J..

[30] Sapsford R., Hodges P. (2001). Contraction of the pelvic floor muscles during abdominal maneavers. Arch. Phys. Med. Rehabil..

[31] Springer B.A., Mielcarek B.J., Nesfield T.K., Teyhen D.S. (2006). Relationships Among Lateral Abdominal Muscles, Gender, Body Mass Index, and Hand Dominance. J. Orthop. Sports Phys. Ther..

[32] Brown S.H.M., McGill S.M. (2010). A comparison of ultrasound and electromyography measures of force and activation to examine the mechanics of abdominal wall contraction. Clin. Biomech..

[33] Abuín-Porras V., de la Cueva-Reguera M., Benavides-Morales P., Ávila-Pérez R., de la Cruz-Torres B., Pareja-Galeano H., Blanco-Morales M., Romero-Morales C. (2019). Comparison of the Abdominal Wall Muscle Thickness in Female Rugby Players Versus Non-Athletic Women: A Cross-Sectional Study. Medicina.

[34] Hodges P.W., Pengel L.H.M., Herbert R.D., Gandevia S.C. (2003). Measurement of muscle contraction with ultrasound imaging. Muscle Nerve.

[35] Seco-Leal M., Da Cuña-Carrera I., González-González Y., Alonso-Calvete A. (2020). Tratamiento de la incontinencia urinaria tras prostatectomía: Una revisión sistemática. Fisioterapia.

[36] Anderson C.A., Omar M.I., Campbell S.E., Hunter K.F., Cody J.D., Glazener C.M.A. (2015). Conservative management for postprostatectomy urinary incontinence. Cochrane Database Syst. Rev..

